# Molecular and cellular limits to somatosensory specificity

**DOI:** 10.1186/1744-8069-4-14

**Published:** 2008-04-18

**Authors:** Carlos Belmonte, Félix Viana

**Affiliations:** 1 Instituto de Neurociencias de Alicante, Universidad Miguel Hernández-CSIC, 03550 San Juan de Alicante, Spain

## Abstract

Animals detect environmental changes through sensory neural mechanisms that enable them to differentiate the quality, intensity and temporal characteristics of stimuli. The 'doctrine of specific nervous energies' postulates that the different sensory modalities experienced by humans result of the activation of specific nervous pathways. Identification of functional classes of sensory receptors provided scientific support to the concept that somatosensory modalities (touch, pain, temperature, kinesthesis) are subserved by separate populations of sensory receptor neurons specialized in detecting innocuous and injurious stimuli of different quality (mechanical forces, temperature, chemical compounds). The identification of receptor proteins activated by different physicochemical stimuli, in particular ion channels of the Transient Receptor Potential (TRP) superfamily, has put forward the concept that specificity of peripheral sensory receptor neurons is determined by their expression of a particular "molecular sensor" that confers to each functional type its selectivity to respond with a discharge of nerve impulses to stimuli of a given quality. Nonetheless, recent experimental data suggest that the various molecular sensors proposed as specific transducer molecules for stimuli of different quality are not as neatly associated with the distinct functional types of sensory receptors as originally proposed. First, many ion channel molecules initially associated to the transduction of only one particular form of energy are also activated by stimuli of different quality, implying a limited degree of specificity in their transducing capacities. Second, molecular sensors associated with a stimulus quality and hence to a sensory receptor type and ultimately to a sensory modality may be concomitantly expressed in sensory receptor neurons functionally defined as specific for another stimulus quality. Finally, activation of voltage gated channels involved primarily in nerve impulse generation can also influence the gating of transducing channels, dramatically modifying their activation profile. Thus, we propose that the capacity exhibited by the different functional types of somatosensory receptor neurons to preferentially detect and encode specific stimuli into a discharge of nerve impulses, appears to result of a characteristic combinatorial expression of different ion channels in each neuronal type that finally determines their transduction and impulse firing properties. Transduction channels don't operate in isolation and their cellular context should also be taken into consideration to fully understand their function. Moreover, the inhomogeneous distribution of transduction and voltage-gated channels at soma, axonal branches and peripheral endings of primary sensory neurons influences the characteristics of the propagated impulse discharge that encodes the properties of the stimulus. Alteration of this concerted operation of ion channels in pathological conditions may underlie the changes in excitability accompanying peripheral sensory neuron injuries.

## Review

The continuous interplay between living organisms and the environment has played a critical role in the evolution and optimization of their sensory capacities. Animals have developed sophisticated neural mechanisms to detect and discriminate environmental changes that enable them to differentiate the quality, location, intensity and temporal characteristics of the various classes of stimuli acting on the body surface and internal organs [1]. Stimuli are detected and encoded by peripheral sensory receptors into trains of nerve impulses that contain the information about those parameters of the stimulus that are particularly relevant for evolutionary success. This neural activity is subsequently processed at different levels of the central nervous system and interpreted finally by the brain as sensations of different perceptual characteristics (i.e. sensory modalities), such as temperature, touch or pain.

In the past few years we have seen a remarkable explosion of studies that are starting to provide a detailed view of the molecules involved in the transduction of the different somatosensory stimuli [2,3]. The quality of the studies and their impact on the field of pain research and sensory neuroscience is unquestionable. However, the physiological interpretation of some of these findings is often, in our opinion, misguided. The main purpose of this review is to alert about some common misconceptions which are rapidly propagating through the literature.

First, we warn about automatically extending the classical concept of specificity of sensory receptor neurons to single transducing molecules. In fact, the picture arising from the study of many putative transduction molecules is complex and rather perplexing considering the extreme polymodality that they exhibit in many cases.

Second, we consider fallacious to assign the functional specificity of a class of sensory afferents to a particular stimulus quality to the presence in their somas of an individual transducing molecule, even in the case when this molecule complies with specificity. The physiological unit of transduction is the sensory ending which, in many cases, coexpress different transduction channels with different activation profiles.

Finally, we caution about the uncritical translation of functional properties of ion channels observed in recombinant systems to the physiological properties of the terminals harbouring those channels. In order to elucidate the coding rules for specific somatosensory stimuli, still much needs to be learned about the transduction process and the properties of native channels and their functional interactions with other molecular elements on nerve terminals.

### Sensations of different modality are conveyed by separate neural pathways

In 1837, Johannes Müller proposed the doctrine of "the specific nervous energies". He postulated that the different modalities of sensation experienced by humans result of the stimulation of specific nervous pathways, now referred to as "labelled lines". These were best activated by a particular type of physical or chemical stimulus, but the perceptual quality of the final sensation remain the same even when that pathway was activated by a different form of energy. In other words, sensation was a function of the active neural pathway not the stimulus. Sensations evoked by stimulation of well-defined sensory organs (vision, audition, smell and taste) were clear examples of this tenet. Those elicited by stimulation of the skin and other structures of the body also appeared to follow the same principle. Thus, on the skin surface, it was possible to identify distinct spots whose natural or electrical stimulation evoked separate tactile, warm or cold sensations, suggesting the existence of specific receptors. These findings were published independently and almost simultaneously in the early 1880s by Magnus Blix, Alfred Goldscheider and Henry Donalson [reviewed by [4]]. Pain was somewhat an exception to this rule and was initially interpreted as the result of "excessive" activation of the other sensory pathways [5]. However, 20 years later, Max von Frey discovered discrete pain points ("Schmerzpunkte") in human skin, extending the concept of "Peripheral Specificity", also termed "Specific Irritability", to all somatosensory modalities [6].

Soon after these proposals were made, researchers tried to establish the anatomical and functional basis of sensory specificity in the somatic system. In the skin, morphologically distinct nerve terminals were identified but the efforts to correlate the anatomy of the various cutaneous receptor types with particular elementary sensory experiences were unsuccessful [7]. With the advent of nerve recording techniques, it was feasible to proof experimentally that peripheral sensory receptors encoded the parameters of the stimulating energy into a discharge of nerve impulses, whose firing frequency reflected with a variable degree of fidelity the characteristics of the stimulus [8]. Furthermore, cutaneous sensory nerve fibers exhibited a marked degree of functional specialization, and different fiber types responding preferentially to touch or to cold and warm temperatures were distinguished [9]. These observations provided a direct proof of the existence, in somatic tissues, of functionally specific peripheral sensory receptors, each of them transducing a particular form of stimulating energy, lending support to the specificity theory of somesthesia [10]. Some of the cutaneous fiber endings were classified as low-threshold mechanoreceptors because they responded preferentially to weak mechanical forces while cold and warm thermoreceptors were primarily activated by moderate cooling or warming of their receptive field. Subsequently, two main populations of nociceptor nerve fibers were distinguished: mechano-nociceptors, excited only by injurious mechanical forces and polymodal nociceptors that also responded to noxious heat, exogenous chemicals and inflammatory mediators [for review see [11]]. More recently, 'silent' (mechanically-insensitive) nociceptors activated only after inflammation develops have been identified [12].

The existence of different subclasses of receptors for low intensity mechanical energy explained the different submodalities of tactile sensations. Cold and warm thermoreceptors would be the origin of sensations of cutaneous cooling and warming while the identification of nociceptors as a functionally distinct group of sensory terminals that responded to a variety of high-intensity injurious stimuli, supported the interpretation that pain, like other somatosensory modalities was subserved by a separate population of receptors specialized in detecting potentially injurious stimuli of different nature [11].

### Transduction molecules are activated by different forms of energy

The search for individual molecular entities associated with the detection of the different stimulating energies was the logical further step in the reasoning that specificity of somatosensory pathways arises at the receptor level. Specificity would be ultimately determined by the presence of a particular "molecular sensor" that confers to each functional class of sensory receptor its selectivity for a particular form of energy. This approach proved to be essentially correct for the visual, olfactory and gustatory sensory pathways, where highly specific transduction molecules, belonging to the family of G protein-coupled receptors, and located respectively in photoreceptor, olfactory and taste receptor cells, were associated in each case with the detection of photons or of particular chemical groups that act as odorants or taste molecules [13]. Activation of each receptor led to the synthesis of cyclic nucleotide second messengers and modulation of the activity of cation channels.

**Figure 1:**
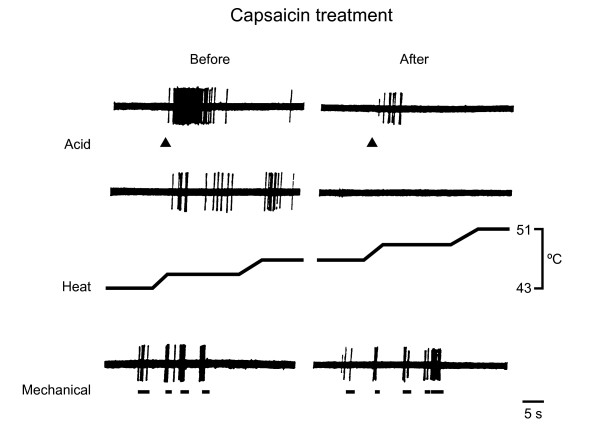
**Separate transduction mechanisms for mechanical and chemical/thermal stimuli in a single polymodal nociceptor terminal**. Example of single fiber recordings from polymodal nociceptive endings innervating the cornea of the cat. Responses to acid, heat and mechanical stimuli, before and after topical application of capsaicin at an inactivating concentration (330 *μ*M). Note the abolition of the response to heat and the marked reduction in the response to local acidic solution (10 mM acetic acid) without affecting the response to mechanical stimulation.

The extrapolation of the same principle to the receptors of somatic and visceral sensory pathways of mammals was experimentally more challenging due to the small size of peripheral sensory nerve terminals. Nonetheless, recordings of single polymodal nociceptor fibers evidenced that sensitivity to heat and acid in an individual fiber could be selectively inactivated without affecting responsiveness to mechanical forces (Figure 1), thus providing an indirect proof that transduction of heat, chemical and mechanical stimuli in individual nociceptive terminals depended on separate molecular entities [14]. Some years later, Cesare and McNaughton discovered a heat-activated cationic current in a subpopulation of nociceptors [15].

**Figure 2:**
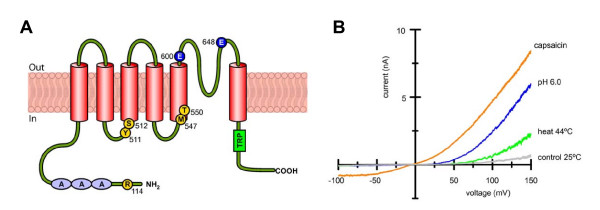
**The capsaicin receptor, a true polymodal sensory receptor molecule**. A. Schematic representation of the topology of a TRPV1 protein subunit. The functional channel is a tetramer formed by the ensemble of four such subunits. Marked in orange are residues influencing capsaicin binding. Marked in blue are two extracellular residues critical for activation by protons. The C-terminal region (shaded green) has been implicated in activation by heat but the critical residues are still unknown. B. Whole-cell I-V relationships of a HEK293 cell transfected with rat TRPV1 showing the activation of currents by low pH (6.0), heat (42°C) and capsaicin (100 nM) (A. Mälkiä, unpublished).

The isolation and subsequent characterization of a functional cDNA encoding a protein involved in the detection of irritant chemicals and heat led to the identification of the first transducing molecule in the mammalian somatosensory system, the 'capsaicin or vanilloid receptor', now named TRPV1 [16]. This membrane receptor is a Ca^2+^-permeable, non-selective cationic channel that belongs to the Transient Receptor Potential (TRP) ion channel superfamily, which were first described in the fruit fly *Drosophila melanogaster *[17]. The functional receptor is probably a tetramer and each subunit is predicted to have 6 transmembrane (TM) spanning domains with a short pore-forming hydrophobic loop between TM domains 5 and 6 (Figure 2). This landmark study showed that the channel was gated by capsaicin [16,18], the pungent component of red peppers long since known as a selective activator of polymodal nociceptor endings [reviewed by [19,20]]. The channel was also opened by temperatures over 43°C and activated by protons (Figure 2) [reviewed by [21]]. It was primarily expressed in small size, unmyelinated neurons identified as putative nociceptors. Therefore TRPV1 was proposed as the molecular substrate for polymodality in these neurons, providing them with the ability to respond to heat and to potentially injurious chemical stimuli causing pain [16,22]. Sustained activation, posttranslational modifications and membrane translocation of TRPV1 channels by a variety of endogenous agents released after injury or inflammation, including bradykinin and endogenous lipids, mediates the sensitizing/excitatory action of polymodal nociceptor neurons during these pathological states [23-28].

**Figure 3:**
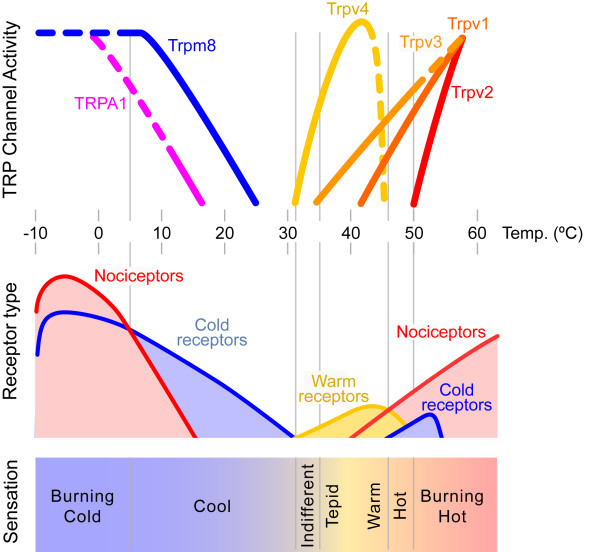
**Hypothetical correspondence between activation of TRP channels, body surface temperature and evoked sensations**. Upper part: Schematic representation of the thermal activation profile of various TRP channels when expressed in recombinant systems. All of them have been located in sensory neurons and/or skin cells. (Adapted from Patapoutian et al. 2003). Middle part: Schematic representation of impulse activity in various cutaneous sensory receptors during application to their receptive fields of temperatures indicated in the thermal scale. Lower part: Quality of sensations evoked in humans by application to the skin of different temperature values. Adapted from Von Frey.

Soon afterwards, other channels of the TRP family were cloned and characterized in heterologous expression systems. Several of them (TRPV2, TRPV3, TRPV4, TRPM8 and TRPA1) were also gated by temperature, with thresholds ranging from 52°C down to 18°C [29-36]. All of them were expressed in primary sensory neurons, or skin keratinocytes, and because they exhibited different temperature thresholds, varying from noxious cold to noxious heat, it was proposed that each of these channel molecules may confer to individual cold and warm thermal receptors and to nociceptors, the ability to respond selectively to innocuous or noxious temperatures across a wide thermal range (Figure 3). These ideas have been summarized in several recent reviews [37-44].

The discovery and characterization of TRPM8, as a transduction channel apparently restricted to a small subpopulation of sensory receptor neurons activated by moderate/non-noxious cold (threshold ~25°C) and by a number of compounds such as menthol, eucalyptol or icilin, known by their ability to induce cold sensations in humans, lend further support to the hypothesis that restricted expression of individual classes of TRP channels in the terminals of subpopulations of primary sensory neurons is the molecular substrate of the specific sensations evoked by each somatosensory stimulus [31,33]. TRPM8 is a non-selective cation channel of the TRPM (melastatine) family with a modest permeability to calcium. The channel is specifically expressed in small diameter trigeminal and dorsal root ganglion neurons in which cooling and menthol evoke inward depolarizing currents and intracellular calcium rises [45-47]. Thus, TRPM8 has been proposed as the cold sensor molecule that confers to peripheral thermoreceptor neurons their specific ability to respond to small temperature reductions, ultimately causing well-defined innocuous cold temperature sensations [41,39]. The characterization of transgenic mice lacking TRPM8 provided direct evidence for the critical role of this channel in cold temperature sensing [48-50].

In contrast to the swift progress in the identification of thermoreceptor proteins, the molecular identity of ion channels involved in the transduction of mechanical stimuli by mammalian somatosensory endings is still uncertain [51]. The fact that mechanosensation requires the concerted function of several proteins acting in an ensemble (transduction apparatus) make these studies particularly challenging. The list of candidate transducer molecules for mechanotransduction also includes several mammalian TRP channels (TRPA1, TRPC1, TRPV4) [reviewed by [52]] and members of the acid-sensing ion channel (ASIC) family [53] that are mammalian homologs of DEG/ENaC channels with a known mechanosensory function in invertebrates. In addition, modulatory roles in mechanotransduction have been assigned to other channels, like two-pore-domain K^+ ^channels [54,55] and P2X purinergic receptors [56].

**Figure 4:**
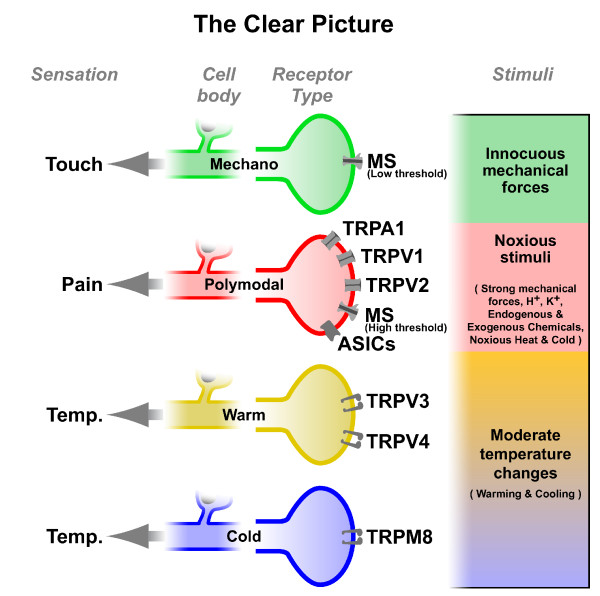
**Molecular basis of somatosensory specificity: The "Clear Picture"**. Schematic representation of various subpopulations of modality specific primary sensory neurons, and the putative 'specific' transduction molecules involved in the detection of the different stimuli.

Thus, the concept of specificity to a particular form of stimulating energy, based on the electrophysiological evidence that sensory afferent fibers preferentially detect and encode a particular type of physical or chemical stimulus, was now extended to a molecular level, meaning that the differential sensitivity of receptor endings resulted of the expression of a particular protein whose presence would be necessary and sufficient to determine the capacity to transduce the specific stimulus into a propagated sensory message and ultimately the modality of the evoked sensation. Figure 4 summarizes schematically these ideas, representing the various subtypes of sensory neurons that respond to specific stimuli, evoking defined sensations and the hypothetical channel molecules in their endings associated to such transduction characteristics. With the exception of TRPV1, recognized from the start as a polymodal receptor, initial descriptions of TRP channel activation have emphasized the selectivity of their activation mechanism, matching this specificity with the sensory modality of a defined subpopulation of sensory fibers [31-33,57,58]. This categorization of transduction channels leads to what could be named in colloquial terms, a "clear picture" of the molecular basis of sensory specificity (Figure 4).

### Gating promiscuity of transduction channels

Nonetheless, a wealth of recent experimental data suggest that specificity of molecular sensors is not as neatly associated with the sensitivity to stimuli of different quality in primary sensory neurons as was originally proposed. First, there is evidence that channel subunits initially associated to the transduction of a single form of energy in a functional class of sensory neuron are also activated by other types of physical and chemical stimuli, suggesting a limited degree of specificity in their transducing capacities. As already indicated, this was quite obvious for TRPV1, recognized since its discovery as a multimodal transduction molecule for chemical and thermal stimuli [16,22], and more recently to mechanical distension of the bladder [59] and to osmotic stimuli in the hypothalamus [60]. In this case, the expression of an N-truncated product of the *Trpv1 *gene gives rise to a functional channel with distinct transducing properties that differ from the full-length TRPV1 protein [60]. This result indicates that alternative splicing of the same gene can add diversity to the coding potential of ion channels derived from a single gene locus.

However, multimodality appears to be also present to a variable degree in many of the members of the TRP channel superfamily implicated in cellular sensing (see Table 1). Unlike for TRPV1, in many of the other TRP channels multimodal gating still lacks a clear physiological interpretation. We will briefly summarize the evidence for several candidate thermotransducers. TRPV2, a relatively close TRPV1 homolog, in addition to heat (threshold >52°C) [29] is activated by cell swelling and membrane stretch [61], and also by growth factors [62] and Δ9-tetrahydrocannabinol [63]. TRPV3, a channel transcribed from a gene adjacent to TRPV1 but lacking sensitivity to capsaicin was identified first as a thermosensitive channel (threshold 34–39°C) [34,35,64]. Subsequent studies identified camphor, a natural medical compound found in the bark of the camphor laurel tree (*Cinnamomum camphora*), and pungent components of oregano and clove as potent agonists of TRPV3 channels [65,66] implying a chemosensory function. Recently, the channel was shown to be directly gated by arachidonic acid and other unsaturated fatty acids [67]. TRPV4 is a channel expressed in a variety of epithelial cells, peripheral sensory ganglia and hypothalamic neurons. It was originally classified as a mechanosensor, activated by hypotonic cell swelling [57,58] and fluid shear stress [68]. However, later studies also established its activation by warm temperatures (threshold ranging from 25–34°C), acidic pH and a variety of chemical compounds [69,30,36,70]. As is the case for other TRPs, mechanical and chemical sensitivity of TRPV4 is strongly modulated by temperature [71]. The gating of TRPV4 by the different agonists (hyposomolality, heat, and many endogenous metabolites) is somewhat special in that separate second messenger pathways are involved in the activation mechanism for a particular stimulus [72].

**Table 1:** Multimodal gating in mammalian somatosensory channels

**Channel**	**Thermal**	**Mechanical**	**Chemical**	**Blockers**
TRPV1	T>42°C	hyper-osmolarity (N-terminal variant), modulates mechanosensitivity	protons, capsaicin, resiniferatoxin, camphor, eugeneol, anandamide, lypooxygenases, 2-APB, clotrimazole, hydroxy-alpha-sanshool	Ruthenium Red (RR), capsazepine, BCTC, DD01050, iodo-resiniferatoxin, SB-452533, Cu:phenantroline
TRPV2	T>52°C	hypo-osmolarity	2-APB, Δ9-THC, probenecid	RR, SKF96365
TRPV3	T>30–39°C		camphor, 6-tert-butyl-m-cresol, carvacrol, eugenol, dihydrocarveol, thymol, carveol and (+)-borneol, 2-APB, arachidonic acid (AA); diphenylboronic anhydride	diphenyltetrahydrofuran, RR,
TRPV4	T>25–35°C	mechanical (sheer stress), hypo-osmolarity	4α-PDD, anandamide, epoxyeicosatrienoic acids, bisandrographolide A	RR
TRPM8	T<23–28°C	membrane tension	menthol, eucalyptol, icilin, WS23, LPC	BCTC, SKF96365, clotrimazole, Cu:phenantroline, intracellular acidification, 2-APB
TRPA1	T<18°C	mechanical	icilin, cinnamaldehyde, mustard oil, allicin, BCTC, 2-APB, 4-hydroxynonenal, hydroxy-alpha-sanshool, acetaldehyde, Δ9-THC, formaldehyde, trinitrophenol, GsMTx-4, methyl p-hydroxybenzoate, menthol, Ca2+	camphor, Gd3+, RR, gentamicin, amiloride, HC-030031, menthol, clorpromazine, AP18
TRPC1		mechanical		Gd3+, La3+, 2-APB
TRPC6		hypo-osmolarity	1-oleolyl-2-acetyl-sn-glycerol	2-APB
ASIC1	cold (positive modulator)	mechanical?	Protons	amiloride, aspirin, Psalmotoxin, A-317567
ASIC2		mechanical	Protons	amiloride, aspirin, A-317567
ASIC3	cold (positive modulator)	mechanical	protons, lactic acid	amiloride, aspirin, APETx2, A-317567
P2X2		mechanical (modulates ATP release)	ATP, UTP	suramin
P2X3		mechanical (modulates ATP release)	ATP, *α*,*β*-meATP	suramin, A-317491, TNP-ATP
TREK1	heat	mechanical	AA, LPC, protons, riluzole, inhalation anesthetics, flufenamic acid	clorpromazine, diltiazem, bupivacaine, fluoxetine, sipatrigine
TREK2	heat	mechanical	AA, LPC, protons, riluzole, inhalation anesthetics, Zn2+, flufenamic acid	Pb2+, diltiazem
TASK1			inhalation anesthetics	protons, Zn2+, hypoxia, anandamide, G*α*q
TASK3			inhalation anesthetics	protons. Zn2+, Ba2+, RR, anandamide, G*α*q

The list of agonists and antagonists cited is extensive but not comprehensive, merely illustrating the degree of polymodality observed in different channels.

Notes:

Agonists for TRPV2 show marked species differences (Neeper et al., 2007).

In the case of TREK and TASK channels, blockers produce an augmentation of excitability while activators reduce it.

The channel subunit TRPA1 was initially proposed as a specific transduction molecule for noxious cold (threshold ~18°C) in peripheral nociceptive neurons [32]. However, recent work has shown that the channel is also gated by various natural pungent compounds such as cinnamaldehyde, allylisothiocyanate and allycin, active ingredients of cinnamon oil, mustard oil and garlic respectively [73-76]. Environmental irritants are also potent activators of TRPA1 [77,78]. While early work on cochlear hair cells also supported a role for TRPA1 in mechanotransduction [79,80], its function as a mechanosensor in the somatosensory system is controversial and unsupported by the behavioural characterization of knockout animals [81,82].

In the case of TRPM8, originally identified as a sensor for mild cold temperatures [31,33], and modulated by a number of chemical compounds, recent work also indicates that it is gated by lysophospholipds and other chemical agents that induce mechanical deformations in the membrane bilayer, suggesting a possible mechanosensitive role [83,84].

The multimodality of TRP channels is a well established concept now and can be traced back to ancient channels (e.g. OCR-2 and OSM-9) in invertebrates [85]. Genetic and molecular studies in *Caenorhabditis elegans *have provided strong evidence that selective activation of TRPV channels to specific stimuli are segregated on different domains of the protein [86]. Similar multimodal gating mechanisms are also frequent among two-pore domain K^+ ^channels, another extended family of ion channels with prominent sensing roles in the somatosensory system (Table 1) [87]. For instance, TREK channels are activated by heat, polyunsaturated free fatty acids including arachidonic acid, phospholipids, volatile anaesthetics, mechanical stretch and intracellular acidification [88-90].

Therefore, many molecular sensors originally ascribed to the response to a given form of energy and primarily associated to the transduction of that stimulus type in the corresponding functional class of sensory neuron, are often also sensitive to physical or chemical stimuli, physiologically linked to a different sensory modality. This gating promiscuity seems to be a common feature of many "transduction" channels and represents in principle an important challenge for an extension of the specificity theory to the molecular level. However, it should be pointed out that for most channels multimodal activation has been observed in heterologous systems. It is unclear whether native channels will show similar properties and in what degree they are activated by different stimuli.

More significant data speaking against extending the specificity hypothesis to transduction molecules were obtained in studies of genetically modified animals. Thus, gene ablation of individual TRPs frequently manifest in a complex sensory phenotype. In TRPV1(-/-) the first deficit identified was a suppression of inflammatory thermal hyperalgesia with no alteration of inflammatory mechanical hyperalgesia [91,92]. However, a more extensive characterization has shown that TRPV1(-/-) mice have clear alterations in thermoregulation [93,94], in the transduction of osmotic responses [60] and mechanical stimuli in the gut [95,96] and bladder [59]. TRPV3(-/-) mice show impaired responses to both, innocuous and noxious heat, suggesting expression in more than one subset of sensory neurons [65]. In the case of TRPV4(-/-) animals, sensory deficits include reduced sensitivity to noxious mechano-osmotic stimuli [97], altered osmotic sensitivity, disturbance of thermal preferences and hearing loss [reviewed by [98,99]]. A complex phenotype is also observed in TRPA1-deficient mice, with alterations in chemical, thermal and mechanical sensitivity [81,82]. The phenotype of TRPM8(-/-) mice, although clearly linked to cold temperature sensing is also heterogeneous. Depending on the behavioural context, the animals can show deficits in responses to unpleasant cold stimuli and deficits in cold-mediated analgesia of inflammatory and neuropathic pain [48-50]. Interpretation of these complex phenotypes in mutant mice may be complicated further by development of compensatory mechanisms which are a common occurrence in conventional global gene inactivation approaches used do far. For example, specific pharmacological inhibition strongly suggests a role of TRPA1 in the sensitization of nociceptors to mechanical stimuli, but compensatory mechanisms completely mask this role in TRPA1 deficient mice [100]

### 'Modality-specific' peripheral sensory neurons also express transduction molecules for different stimuli

Not only are many transducing molecules multimodal for different energy forms. Several of the molecular sensors associated to a stimulus and hence to a sensory modality, are concomitantly expressed in sensory neurons that are functionally defined as specific for another stimulus quality, thus resembling pain-signaling, polymodal nociceptor terminals. These terminals, in addition to TRPV1 possess stretch-activated channels of still unknown molecular nature, which mediate their sensibility to noxious mechanical forces, and ASIC channels that contribute to their response to acidic pH [101,102]. This is also the case of cold-sensitive thermoreceptor neurons expressing TRPM8; about 50% of them are also activated by capsaicin, a specific activator of TRPV1 channels [39,103-107]. This dual expression possibly explains the responsiveness of a part of cold sensitive terminals to heat and capsaicin [108]. Indeed, careful experimental scrutiny shows that many thermosensitive afferents can be activated by low and high temperatures, a finding that could explain several interesting perceptual phenomena like paradoxical cold or synthetic heat [reviewed by [109]]. It is also well known that a fraction of low threshold cutaneous mechanoreceptors are activated by cooling [110-114] thus suggesting that they possess some of the transducing mechanisms for temperature present in specific thermoreceptor neurons.

### 'Modality-specific' peripheral sensory neurons express multiple transduction molecules for the same type of stimulus

Additional evidence illustrating the biological complexity in the organization of transducing processes in the somatosensory system stems from the observation that separate molecular sensors detecting the same type of physical or chemical stimulus may co-exist in an individual sensory receptor type. This overlap was soon realised upon phenotypic characterization of null mutants for single transduction molecules. For instance, the persistence of sensitivity to heat in polymodal sensory endings of TRPV1(-/-) mice has been explained by co-expression in polymodal neurons of other heat-sensitive TRP channels [91,92,115]. Specifically, TRPV3 [35] and TRPV2 [116,117] channels have been shown to co-express, and even form heteromultimers, with TRPV1 subunits. Also, the recent development of potent TRPV1 antagonists has illustrated their lack of effect on acute thermosensory responses [118], strongly suggesting the overlap between peripheral thermosensitive mechanisms operating *in vivo*. Expression of transducers is not the only factor determining the physiological response of afferents to peripheral stimuli. Of note, the variable sensitivity to noxious heat among many different strains of mice is mediated by the differential expression of the CGRP gene, further illustrating the complex interactions between thermal transducers and other signal modulators *in vivo *[119].

Similarly, the excitatory effects of protons on nociceptors are mediated by multiple channel subunits including TRPV1, ASICs, and TASK potassium channels [101,102,120-123]. Targeted disruption of individual subunits produces variable deficits in the response to low pH in different classes of nociceptors [91,124] with no obvious deficit in some of them [124], suggesting a significant overlap in the pattern of expression of these transduction channels [125].

In neurons sensitive to non-noxious cold, temperature reductions open TRPM8 channels [31,46,47,126] but also close thermosensitive background K^+ ^channels [104,127,128] and, as commented below, the final depolarization induced by cold is the result of the summation in the same neuron of separate transduction mechanisms activated by the specific stimulus. Consistent with this view, cold-evoked responses are not abrogated in TRPM8(-/-) mice; the remaining cold-sensitivity varied in the three studies [48-50]. A large fraction of neurons known to express TRPM8 remain cold sensitive even after genetic ablation of TRPM8 [129]. Additional evidence for this concept of redundancy in transduction molecules for a stimulus modality has been obtained with pharmacological approaches. In a recent study, performed in cold-sensitive nerve terminals of the cornea, selective pharmacological blockade of TRPM8 channel function abolished the activation by menthol of the terminals but left largely unaffected their ability to be excited by cold stimuli [47]. The presence in the same neuron of multiple thermal sensors is also evident in developing sensory neurons, that can respond massively to cooling even before detectable expression of known thermoTRP channels [130].

It is tempting to speculate teleologically that this functional overlap provides sensory neurons with a larger operating range than that offered by a single transduction channel and additionally offers the possibility of different responses to external and internal modulators, adding flexibility to the system.

### Limited 'molecular' specificity of sensory receptor terminals

**Figure 5:**
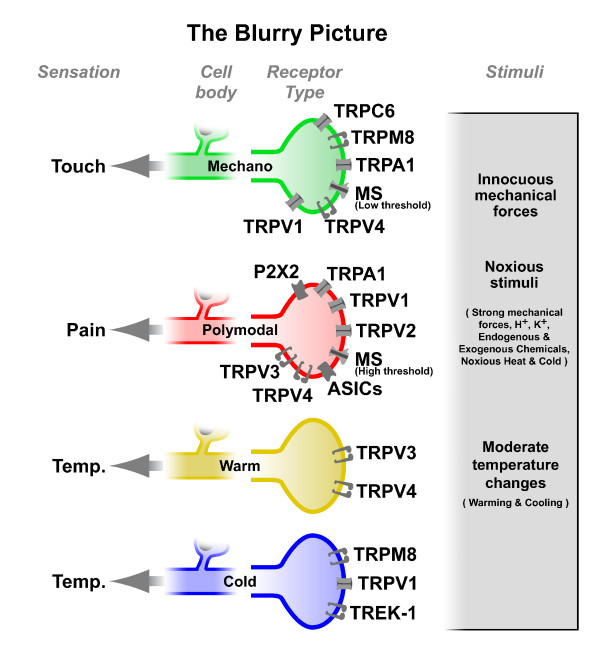
**Molecular basis of somatosensory specificity: The "Blurry Picture"**. Schematic representation of various subpopulations of modality specific primary sensory neurons, and of some of the putative transduction molecules that could be involved in their detection capacities for different stimuli. Data for the different channels potentially involved in nociception have been lumped together into an oversimplified model of polymodal neuron. Also, the stimuli refer to the preferred stimulus for each class of neuron but does not exclude the activation by other types.

Taken together, the available experimental evidence speaks against the generalization that in the somatosensory system there is a specific molecule for every stimulus type and a specific class of neuron for each transducing molecule. Rather, the capacity exhibited by the different types of primary sensory neurons to preferentially detect and encode the specific stimuli into a discharge of nerve impulses appears to depend on a characteristic combination in each neuronal class of different transduction mechanisms provided by a combinatorial expression of molecular sensors. We see a more "blurry picture", schematized in Figure 5.

The limited specificity of transduction molecules and of single primary sensory neurons, contributes to explain at the peripheral level some perplexing aspects of the sensations experienced when two stimuli of different quality act simultaneously on a particular sensory pathway. Cold sensitivity of low threshold mechanoreceptors is apparently the origin of the 'Weber paradox' or Silver Thaler Illusion (i.e. the feeling that cold objects feel heavier than when they are warm [131]). The presence of TRPV1 in a fraction of innocuous cold receptor neurons [107] may be the explanation for the sensation of cold experienced when heat is applied to cutaneous cold spots (von Frey, 1895) and for the response of a fraction of cold-sensitive fibers to heating over 42–45°C [132]. These remarkable psychophysical phenomena may also originate centrally, by the convergence of sensory information of different modality, as exemplified by the thermal grill illusion [133].

### Response of nerve terminals is shaped by the interaction of transduction channels with other molecular elements

Additionally, we would like to emphasize that the processes of transduction and encoding of the stimulus take place in the same physical structure, the peripheral nerve ending, where both processes interact. Thus, currents generated by subthreshold and supratherhold activation of voltage gated channels involved in nerve impulse generation can influence, directly or indirectly, the gating of transducing channels. In this respect, it is important to note that many TRP channels are also voltage sensitive [134,135], and the interaction of chemical and thermal stimuli with the voltage-sensing mechanism plays a key role in their gating mechanism [136-140].

**Figure 6:**
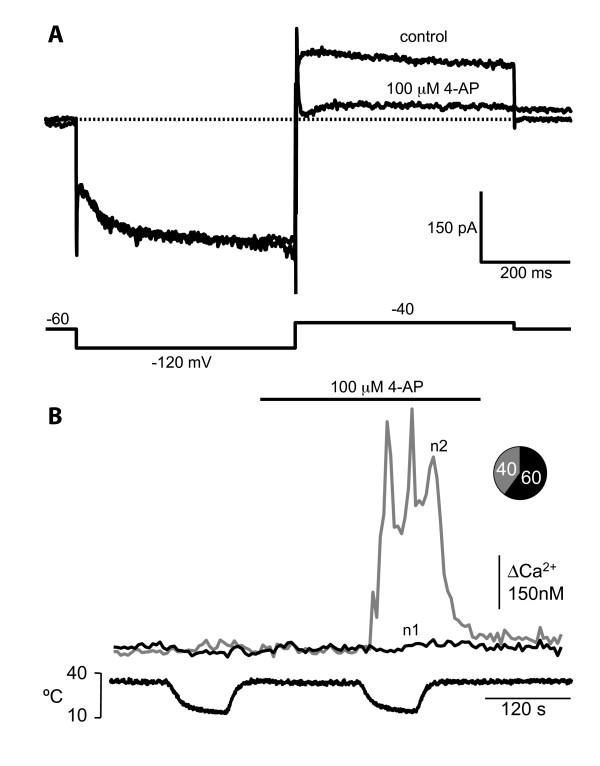
**Phenotypic transformation of sensory neuron by K^+ ^channel blockade**. A. Blocking effect of 100 *μ*M 4-AP on a slowly-inactivating K^+ ^current that is responsible for preventing the response to cooling in cold insensitive trigeminal ganglion neurons. B. Simultaneous recording of [Ca^2+^]_i _and bath temperature in two trigeminal sensory neurons. The application of 100 *μ*M 4-AP unmasked a [Ca^2+^]_i _response in n2 during the second cooling step, transforming a cold-insensitive neuron into a cold-sensitive one. Adapted from Viana et al., 2002.

A clear example of the functional interaction between voltage-gated and transducer channels is exemplified in Figure 6. In a subpopulation of primary sensory neurons that do not respond to a cooling stimulus, application of low concentrations of 4-aminopyridine (4-AP), a K^+ ^channel blocker, results in the appearance of a novel excitatory response to cooling [104,141]. The cellular mechanism that gives rise to this emerging phenotype is the blockade of a slow transient outward K^+ ^current that is acting as an excitability break [104]. In other words, in these neurons the presence of such current prevents the depolarizing effect exerted by an excitatory cold-sensitive conductance restricting their activation profile [104].

An additional example of subtractive interactions between two thermosensitive channels has been found recently in C-type nociceptors. Many of these neurons co-express the background K^+ ^channel TREK-1 and TRPV1. The activity of both channels is augmented by heat [89,16] but, while activation of TREK-1 acts as a brake, TRPV1 drives the excitation. Accordingly, heat-sensitive fibers in TREK1(-/-) animals show a much enhanced response to warm stimuli compared to wildtype animals [87].

Another dramatic example on functional interactions between channels in the peripheral nervous system comes from studies on mutations in the sodium channel Nav1.7 which lead to primary erythromelalgia [142]. Remarkably, a single misense mutation (F1449V) can produce opposing phenotypes in two classes of peripheral neurons where Nav1.7 is expressed, hyperexcitability in DRG neurons and hypoexcitability in sympathetic neurons [143]. The differences depend on the co-expression of Nav1.8 (in DRG) or lack of it (in sympathetic neurons) with Nav1.7.

Direct evidence for functional interactions between transduction and encoding ion channels residing on the same nerve terminal is limited, primarily due to the extreme difficulties in recording from somatosensory endings. In corneal cold receptor terminals, the degree of inactivation of sodium channels by the variable membrane potential determines their excitability [144]. In cutaneous sensory terminals, receptor potentials generated by cold interact with TTX-insensitive NaV1.8 channels and are a critical element of their selective excitability during cooling [145]. This means that expression of a particular transducer (e.g. a cold sensor) could be general but transmission of a propagated cold sensory message would be restricted to specific subpopulations of nerve fibers by its combinatorial expression with voltage-gated channels (i.e. K^+ ^and Na^+ ^channels [104,145].

The cellular environment can also play an important role in shaping the transducing capacities of ion channels. In the case of TRPM8, it is clear that native channels have temperature activation thresholds that are significantly warmer than heterologously expressed channels [146,47,147]. Thus, mean static discharge of many trigeminal cold receptors peaks at 28–30°C, temperatures higher than the threshold of recombinant TRPM8 channels [148]. The intrinsic signals modulating the shift in thermal sensitivity of endogenous TRPM8 channels are currently unknown. TRPM8 activity depends on the presence of PIP_2 _[149-151]. It is also worth noting that TRPM8 responses to cold are abolished by phospholipase A2 (PLA2) inhibitors and sensitized by PLA2 products such as lysophospholipids (LPLs) (lysophosphatidylcholine, lysophosphatidylinositol, and lysophosphatidylserine) [84]. Another example of context-dependent function is observed in TRPV4. In artificial expression systems, TRPV channels are activated by hypotonic cell swelling [57,58,72]. In contrast, expression of TRPV4 in OSM-9 mutant worms can rescue the hypertonicity avoidance behaviour [152].

### Concluding remarks

The evidence outlined above is contrary to the temptation in many contemporary neuroscientists to assign particular sensations to single molecular entities and illustrates the convenience of avoiding an oversimplified scheme of the mechanisms used by the various sensory receptors to detect a particular form of energy to finally evoke a specific perceptual experience. Sensory transduction channels don't operate in isolation but rather show important direct and indirect interactions with other ion channels and signalling molecules. Thus, we caution against the tendency to disregard cellular context in the investigations on their function.

Moreover, in any given peripheral sensory neuron type, the presence of a specific molecular structure responding to the same physical or chemical perturbations that act as the natural stimuli for its receptor terminals, does not prove unequivocally that this is the sole or even the principal transduction mechanism used by that particular receptor neuron. As the activation profiles of single molecular sensors do not appear to recapitulate the transduction capacities of single nerve terminals, it seems more likely that discrimination capacity for the adequate stimulus is dictated by a more complex interaction between specific transduction molecules and other ion channel proteins. Their presence and inhomogeneous distribution at soma, axon and peripheral endings will ultimately determine the characteristics of the propagated impulse discharge that encodes the properties of the stimulus, conferring functional specificity to the various types of sensory receptor neurons.

Thus, although the experimental evidence that primary sensory neurons represent "labelled lines" responding preferentially to an energy form remains strong today, an extrapolation of the classical specificity theory to the molecular level in the somatosensory system lacks, at present, solid experimental support. Moreover, a rigid extension of the specificity theory to every mechanism involved in stimulus detection conflicts with the complexity and multimodal characteristics of many peripheral receptor responses to thermal, mechanical and chemical stimuli. Very likely, this is also applicable to higher levels of the nervous system [153], where the presence of 'cross talk' between sensory pathways adds variability and richness to the different conscious sensations finally elicited by a complex multifaceted environment.

It can be expected that the combined efforts of many laboratories approaching the problem at different conceptual levels and applying a range of powerful techniques (gene manipulation, cell imaging, electrophysiology, pharmacology, behavioural analysis) will lead to rapid progress in deciphering the intimacies governing the transduction mechanisms of ambient stimuli at mammalian somatosensory endings.

## Competing interests

The authors declare that they have no competing interests.
